# VIDA-Nursing v1.0: immersive virtual reality in vacuum blood collection among adults*

**DOI:** 10.1590/1518-8345.3685.3263

**Published:** 2020-06-01

**Authors:** Valtuir Duarte De Souza-Junior, Isabel Amélia Costa Mendes, Romero Tori, Leonardo Prates Marques, Felipe Kenzo Kusakawa Mashuda, Leonardo Akira Fattore Hirano, Simone De Godoy

**Affiliations:** 1 Universidade de São Paulo, Escola de Enfermagem de Ribeirão Preto, PAHO/WHO Collaborating Centre for Nursing Research Development, Ribeirão Preto, SP, Brazil.; 2 Fundação Hemominas, Hemocentro Regional de Uberaba, Uberaba, MG, Brazil.; 3 Universidade de São Paulo, Escola Politécnica, São Paulo, SP, Brazil.; 4 Scholarship holder at the Fundação de Amparo à Pesquisa do Estado de São Paulo (FAPESP), Brazil.; 5 Scholarship holder at the Conselho Nacional de Desenvolvimento Científico e Tecnológico (CNPq), Brazil.

**Keywords:** Virtual Reality, Simulation, Simulation Training, Blood Specimen Collection, Technological Development and Innovation Projects, Nursing, Realidade Virtual, Simulação, Treinamento por Simulação, Coleta de Amostras Sanguíneas, Projetos de Desenvolvimento Tecnológico e Inovação, Enfermagem, Realidad Virtual, Simulación, Entrenamiento Simulado, Recolección de Muestras de Sangre, Proyectos de Desarrollo Tecnológico e Innovación, Enfermería

## Abstract

**Objective:**

to develop and validate the first immersive virtual reality simulation addressing vacuum blood collection in adult patients - VIDA-Nursing v1.0.

**Method:**

methodological study to validate 14 steps of the vacuum blood collection procedure in adults, designed to develop the immersive virtual reality simulator VIDA-Nursing v1.0. It was assessed by 15 health workers and 15 nursing undergraduate students in terms of visual, interactive, movement simulation reality, teaching and user-friendly aspects.

**Results:**

the workers considered 79.6% of the items to be valid, while the students considered 66.7% of the items valid; most of the demands can be implemented in the system by improving future versions.

**Conclusion:**

the simulator was considered a promising and innovative tool to teach vacuum blood collection in adults as it can be combined with other resources currently used to introduce this topic and technique in the education of undergraduate nursing students.

## Abstract (pt)

**Objetivo:**

desenvolver e validar a primeira versão do simulador de realidade virtual imersiva no procedimento de coleta de sangue a vácuo no paciente adulto - VIDA-Enfermagem v1.0.

**Método:**

estudo com delineamento metodológico para validar 14 etapas do procedimento de coleta de sangue a vácuo no adulto, projetadas para o desenvolvimento do simulador de realidade virtual imersiva VIDA-Enfermagem v1.0, o qual foi avaliado por 15 profissionais da saúde e 15 graduandos de enfermagem em relação aos aspectos visual, interativo, realidade de simulação do movimento, pedagógico e esforço de utilização.

**Resultados:**

de maneira geral foram considerados válidos 79,6% dos itens avaliados pelos profissionais e 66,7% dos itens avaliados pelos graduandos, sendo que a maioria das necessidades de melhorias do sistema é passível de correção no incremento das próximas versões.

**Conclusão:**

o simulador foi considerado como ferramenta promissora e inovadora para o ensino da coleta de sangue a vácuo no adulto, enquanto estratégia a ser combinada com recursos utilizados atualmente na educação de graduandos de enfermagem que estão iniciando o estudo da temática e da técnica.

## Abstract (es)

**Objetivo:**

desarrollar y validar la primera versión del simulador de realidad virtual inmersivo en el procedimiento de recolección de vacío de sangre en pacientes adultos: VIDA-Enfermería v1.0.

**Método:**

estudio con diseño metodológico para validar 14 pasos del procedimiento de extracción de sangre al vacío en adultos, diseñado para el desarrollo del simulador inmersivo de realidad virtual VIDA-Enfermería v1.0, que fue evaluado por 15 profesionales de la salud y 15 estudiantes universitarios de enfermería con relación a los aspectos visual, interactiva, realidad de movimiento, pedagógico y esfuerzo de uso.

**Resultados:**

en general, el 79.6% de los ítems evaluados por los profesionales y el 66.7% de los ítems evaluados por los estudiantes de pregrado se consideraron válidos, y la mayoría de las necesidades de mejoras del sistema están sujetas a corrección en el incremento de las próximas versiones.

**Conclusión:**

el simulador fue considerado como una herramienta prometedora e innovadora para enseñar la extracción de sangre al vacío en adultos, como una estrategia que se combina con los recursos utilizados actualmente en la educación de estudiantes de enfermería que están comenzando a estudiar el tema y la técnica.

## Introduction

Peripheral venipuncture is defined as the insertion of devices through a peripheral vein to access one’s bloodstream. It is a common, however, complex procedure performed in health care that demands competent workers to perform it^(^ 1 ^-^ 3 ^)^. A peripheral venipuncture is an essential stage in procedures for various purposes, among which intravenous therapy and the collection of blood samples for laboratory exams.

Inappropriately performing this stage may expose patients to site complications such as phlebitis, infiltration and/or hematoma and, when associated with incorrect procedures performed in other stages, complications may be systemic, such as thrombophlebitis and bloodstream infections, which are more frequently associated with the use of intravenous devices^(^ 1 ^-^ 2 ^,^ 4 ^-^ 7 ^)^.

A peripheral venous puncture inadequately performed may also compromise the results of laboratory exams, which is one of the major aspects to be continuously addressed by the staff in the pre-analytical phase of blood collection^(^ 8 ^)^ and may reflect on improper conducts in a patient’s treatment^(^ 9 ^-^ 11 ^)^.

There are many technologies to develop skills and competencies related to the collection of blood samples and peripheral venous catheterization that include conventional arm simulators, models with latex veins that can be attached with straps over a human arm, and non-immersive virtual reality simulators.

Various teaching institutions have increasingly incorporated the use of simulators in teaching strategies. Simulators can be used to develop technical skills, interpersonal relationships, promote specific competencies or problem-solving skills while various types of simulators and environments, including the virtual environment, are employed^(^ 12 ^)^. In this context, virtual refers to environments or elements synthesized by digital devices with the possibility to be immaterially replicated^(^ 13 ^)^.

Technological development has enabled interaction between person-machine to become increasingly advanced, facilitating the development of more realistic virtual environments. From this perspective, virtual reality (VR) promotes interactive and motivating simulators. VR is an advanced interface generated by computer-performed applications, through which users interact in real-time, stimulating the senses with elements of a three-dimensional environment such as visualization, movement, hearing and/or touch^(^ 14 ^)^.

VR simulations have proven to be feasible, as reported by recent international studies, with important results both to support human resources training and the treatment of patients. There are two clinical trials reporting results in the treatment of patients^(^ 15 ^-^ 16 ^)^ and five clinical trials addressing the training of human resources^(^ 17 ^-^ 21 ^)^. There are also other types of study designs conducted in the field of human resources training^(^ 22 ^-^ 23 ^)^.

Fifteen meta-analyses report VR simulators intended to support the treatment of patients^(^ 24 ^-^ 38 ^)^, while two other meta-analyses report its use in the training of human resources^(^ 39 ^-^ 40 ^)^. In general, the studies present promising results in the use of VR simulations. The field of training focused on medical procedures in the surgical field in general and in laparoscopic surgery, hysteroscopy, mastoidectomy, and sutures.

There is, however, little evidence presented in the literature regarding the use of VR simulations in the teaching of nursing and we believe this technology can contribute with innovations in the training of human resources in health, mainly specialties, in which, lack of knowledge and skills directly affects the safety and integrity of patients.

Hence, considering that peripheral blood collection is one of the first invasive procedures commonly taught during the training of human resources in nursing, we opted for developing an immersive virtual reality simulator applied to the context of vacuum blood collection in adult patients.

Therefore, this study’s objective was to develop and validate the first immersive virtual reality simulation addressing vacuum blood collection in adult patients - VIDA-Nursing v1.0.

## Method

Study with methodological design to develop and validate the 14 stages of the vacuum blood collection procedure performed in adult patients using the immersive virtual reality simulator VIDA-Nursing v1.0. The name refers to VIDA-Odonto^(^ 41 ^)^, which is built upon the environment VIDA (Virtual Interactive Distance-Learning on Anatomy). The study received approval from the Institutional Review Board at the University of São Paulo at Ribeirão Preto, College of Nursing (EERP-USP), under CAAE 63058516.8.0000.5393.

Criteria used to develop the simulator were based on evidence reported in the literature concerning the vacuum blood collection procedure in adult patients^(^ 8 ^,^ 42 ^-^ 43 ^)^. Given the technological complexity of developing an immersive virtual reality simulator to train individuals for an invasive procedure, basic requirements were established to be applied in the short term, to test and verify the system feasibility, to be later complemented at the medium and long terms.

In total, 16 requirements were listed for the procedure to be performed in the short term, namely: 1- Apply tourniquet on patient’s arm; 2- Clean the venipuncture site with cotton soaked with alcohol at 70%; 3- Remove cap from needle; 4- Use non-dominant hand to secure the vein; 5- Puncture the patient’s vein with bevel facing upwards, at an oblique angle of 10º to 30º, compatible with the vein’s depth; 6- Introduce the vacuum tube into the adapter; 7- Verify that blood is flowing into the tube; 8- Release and remove the tourniquet from patient’s arm; 9- Wait for the vacuum tube to be filled; 10- Remove the blood-filled tube from the adapter; 11- Homogenize the blood sample by making gentle inversion movement, from 5 to 10 times; 12- Bring dry cotton near the site where needle is piercing the arm; 13- Remove the needle; 14- Place the cotton and compress the site; 15- Activate safety device to protect the needle; 16- Place the intermediary device on the tray. Important to note that requirements 3 and 15 had not been included in the first version given the difficulty in reproducing these movements in a virtual environment, thus these will be implemented in the simulator’s second version.

The team was composed of eight people working directly in the development of the first version of VIDA-Nursing v1.0: one undergraduate nursing student, two electrical engineering graduate students, one mechanical engineer, one electrical engineer, and three nurses.

The following were used for storing, sharing, managing and developing the modeling and programming of the environment and virtual objects: Unreal 4.18 (Game Engine); Visual Studio Community 2017 (Image editor); Blender 2.79 (3D modeler); GitHub (Online project and version management service); Git for Windows 2.16.1, 64-bit (Local management of Projects and Versions); TortoiseGit 2.6.0, 64-bit for Windows (Graphical Interface for Git); Inventor - AutoDesk 2018 (3D CAD Modeler); AutoCad - AutoDesk 2018 (3D CAD Modeler). The programs were run on the equipment: Computer Dell model XPS 8920; Leap Motion (Gesture Sensor); Oculus Rift (-Head Mounted Display)+Touch Virtual Reality System (Control). The character used as a patient was a 3D model provided by Adobe^©(^ 44 ^)^ free of charge to create, manipulate, and animate virtual design projects.

Content Validity Index (CVI) was used to validate the simulator. The CVI of the individual items is calculated to measure the proportion of agreement among the panel of judges when assessing a measuring instrument. The calculation used for each item was CVI=number of responses 1 + 2 divided by the total number of answers, considering a minimum agreement of 80% for each item assessed^(^ 45 ^-^ 46 ^)^.

The form used to validate the simulator addressed the following: Visual, Interactive, Movement Simulation Reality, Teaching, and User friendliness aspects. The authors developed the form and three workers with clinical and teaching experience in the procedure addressed here, validated it both in terms of face and content validity.

After taking part in the simulation, the participants rated their level of agreement with the statements that concerned the items presented in the assessment form, on a Likert scale ranging from strongly agree, neither agree nor disagree, disagree and strongly disagree.

Face and content validation was conducted by two groups: group 1 was composed of 15 health workers who mastered the topic and the vacuum blood collection procedure performed in adult patients and were recruited for being professors of theoretical-practical courses addressing this content in the institution where data were collected, and; group 2 composed of 15 undergraduate nursing students who had already performed the vacuum blood collection procedure in a simulated situation. None of the participants in the groups had had any previous contact with this simulator.

Data were collected from October 16^th^ to 23^rd^ 2018. Each group was invited for the simulation in the VIDA-Nursing v1.0 simulator installed in the laboratory of the Grupo de Estudos e Pesquisas em Comunicação no Processo de Enfermagem - GEPECOPEn [Study and Research Group on Communication in the Nursing Process] at EERP-USP. The environment was prepared for the participants to receive information regarding the study, sign a free and informed consent form, take part in the simulation, and complete the forms concerning characterization and assessment of the simulator, so that, their performance could be assessed. The monitor screen was video recorded during the simulation to support analysis and clarify potential doubts regarding the participants’ performance.

The theoretical framework used to support the development of the simulator was provided by Skinner. Skinner’s study on operant conditioning behavior has been used in educational games, entertainment games, and virtual simulations used in teaching^(^ 47 ^-^ 49 ^)^. Operant conditioning refers to an organism’s response through differential reinforcement of successive approximations, in which a response generates a consequence, which affects the probability of it occurring again in the future^(^ 50 ^)^.

Data were coded and typed twice in Excel spreadsheets and then exported and analyzed in SPSS (Statistical Package for Social Science) version 22.0. Descriptive statistics were used, including an analysis of frequency and percentage, and CVI with a minimum agreement of 80% among the items assessed. The participants’ suggestions were also addressed to be implemented in the following versions.

## Results

The workers’ ages ranged from 22 and 53 years old, with an average of 32.7 years old, while most were women (80%). About graduate programs, ten (66.7%) had a specialization; nine (60%) had a Master’s degree; seven (46.7%) had a Doctoral degree, and one (6.7%) had attended a post-doctorate program. In terms of experience in vacuum blood collection, only one (6.7%) reported no specific experience with the vacuum technique; 13 (86.7%) had already collected blood samples using a vacuum tube in an adult anatomical dummy, and three (20%) reported some experience using a virtual reality simulator. Among the experiences reported, one referred to entertaining games using a head-mounted display (HMD) and two reported using a Virtual IV^®^ simulator as a teaching strategy.

The ages of the undergraduate nursing students ranged from 20 to 26 years old, 22.3 years old on average, while 11 were women (73.3%). Concerning the undergraduate program, five (33.3%) were from the Nursing Teaching Diploma program while 10 (66.7%) were from the Bachelor’s Nursing program. Regarding their previous experience with the vacuum blood collection procedure, 11 (73.3%) had previously drawn blood samples using a vacuum tube from adult patients, 15 (100%) had performed vacuum blood collection using an anatomical adult dummy, and eight (53.3%) reported the use of a virtual reality simulator. The experiences reported with a virtual reality simulator included the use of entertaining games using HMD, while seven had used it in a teaching situation. Four of these had experienced IV^®^ Virtual simulator addressing the subject at hand, two had experienced a virtual learning environment in the neonatal field, and one had used a car simulator provided in a driver training school.

The Content Validity Indexes obtained by the workers and college students in aspects presented by the VIDA-Nursing simulator v.1.0 were, respectively:

Visual Aspects - Q1- In general, the virtual objects have a realistic appearance (1.0 and 0.9); Each of the following objects are realistic in regard to their appearance: Q2- Chair (1.0 and 0.8); Q3- Patient (1.0 and 0.9); Q4- Bench (0.9); Q5- Tray (0.9 and 0.8); Q6- Intermediary device (1.0 and 0.9); Q7- Needle (1.0 and 0.7); Q8- Dry cotton (0.9 and 0.7); Q9- Cotton soaked with alcohol (0.8 and 0.5); Q10- Tourniquet (1.0 and 0.7); Q11- Hands (0.9). The virtual objects are realistic in terms of scale: (relationship among the objects’ dimensions) Q12- Chair (0.9); Q13- Patient (0.8 and 0.9); Q14- Bench (0.9); Q15- Tray (0.9 and 0.7); Q16- Intermediary device (0.9 and 0.8); Q17- Needle (0.9 and 0.8); Q18- Dry cotton (0.9); Q19- Cotton soaked with alcohol (0.9); Q20- Tourniquet (0,9); Q21- Hands (0.9 and 1.0). The virtual objects are realistic in regard to the model’s position in the environment: Q22- Chair (1.0 and 0.9); Q23- Patient (0.9); Q24- Bench (0.9); Q25- Tray (0.9); Q26- Intermediary device (1.0 and 0.9); Q27- Needle (1.0); Q28- Dry cotton (1.0 and 0.9); Q29- Cotton soaked with alcohol (1.0 and 0.9); Q30- Tourniquet (0.9); Q31- Hands (0.9 and 1.0); Q32- The number of virtual objects in the environment is sufficient for the proposed simulation (0.9 and 1.0);
Interactive Aspects - Q33- The device movements were precise (relationship between movement, speed of intermediary device/virtual needle and movement) (0.3); Q34- The space available to perform the puncture is sufficient (amplitude of movement during manipulation) (0.7 and 0.9); Q35- Visualization of the environment (immersion environment) is sufficient for the simulation experience (0.9); Q36- The environment where hands actually move to perform the puncture (hands are free) is sufficient for the simulation experience (0.9);
Movement Simulation Reality Aspect - Q37- Place tourniquet on arm (0.5); Q38- Skin asepsis (1.0 and 0.8); Q39- Tight the skin with dominant hand (0.8 and 0.5); Q40- Venipuncture (0.7 and 0.2); Q41- Insert the vacuum tube into the adapter (0.7); Q42- Blood flowing into the tube (0.9); Q43- Release tourniquet from patient’s arm (0.6 and 0.5); Q44- Removal of blood-filled tube from the adapter (0.5); Q45- Homogenization of blood sample (0.7 and 0.9); Q46- Needle removal (0.6 and 0.5); Q47- Compression of the puncture site with cotton (0.9 and 0.7); Q48- Placement of the intermediary device on the tray (0.9 and 0.7);
Teaching Aspect - Q49- An immersive virtual simulation can be a tool to teach the peripheral venipuncture to collect blood samples using a vacuum tube (1.0); Q50- Research addressing immersive virtual simulation can contribute to teaching in the nursing field (1.0); Q51- The immersive environment during simulation contributed to learning the procedure of peripheral venipuncture to collect blood samples using a vacuum tube (1.0);
User-friendly Aspect - Q52- The simulator is user-friendly (0.7); Q53- The simulator is difficult to use (0.4 and 0.3); Q54- The simulator is tiresome (0.1 and 0.3).

Data concerning the participants’ occupation and performance are presented in Figures 1 to 3.

**Figure 1:** Formal education and occupation of the workers who took part in the VIDA-Nursing v.1.0 Simulation. Ribeirão Preto, SP, Brazil, 2018

	Background	Time since graduation	Years in current occupation	Previous occupation
**1**	Nurse	30 years	Teaching (12 years)	Care delivery (18 years)
**2**	Nurse	8 years	Graduate student	Medical Clinic (3 years)
**3**	Biochemical pharmacist	6 years	Public employee (8 years)	Teaching (10 years)
**4**	Nurse	15 years	Teaching (2 years)	Hospital Care (15 years); Intensive Care (6 years)
**5**	Nurse	3 years	Graduate student	Primary healthcare (11 months); Urgency/Trauma (2 years); Intensive Care (6 months)
**6**	Nurse	12 years	Graduate student	Care delivery (8 years); Teaching (4 years)
**7**	Nurse	6 years	Graduate student; Teaching (2 years)	Pre-hospital care (3 years); Intensive Care (2 years)
**8**	Nurse	7 years	Graduate student; Teaching-Administrative (3 years)	Primary Healthcare (2 years)
**9**	Nurse	16 years	Teaching (9 years)	Care delivery (7 years)
**10**	Nurse	15 years	Teaching (12 years)	-
**11**	Nurse	7 years	Teaching in Family Health (2 years)	Head of a Primary Healthcare Unit (1 year)
**12**	Nurse	5 years	Graduate student	Care delivery (1 year); Deft interpreter (5 years)
**13**	Nurse	4 years	Teaching (2 months); Care delivery (1 month)	-
**14**	Nurse	5 years	Graduate student	Teaching (4 months)
**15**	Nurse	3 years	Graduate student	-

**Figure 2:** Performance of the workers who took part in the VIDA-Nursing v.1.0 Simulation. Ribeirão Preto, SP, Brazil, 2018

Performance	1	2	3	4	5	6	7	8	9	10	11	12	13	14	15	Total
Duration of 1^st^ environment	00'50''	01'15''	02'25''	01'38''	01'21''	02'34''	01'29''	05'10''	01'52''	02'25''	03'20''	02'20''	02'44''	01'24''	01'57''	32'44'' (M*-02'11'')
Duration of simulation	16'38''	07'38''	15'00''	20'10''	23'53''	18'21''	08'58''	28'49''	22'38''	11'09''	15'48''	16'12''	19'28''	10'48''	13'47''	249'17'' (M*-16'37'')
Duration/first attempt	16'28'' /11^th^	07'23'' /2^nd^	14'31'' /5^th^	06'58'' /2^nd^	20'36'' /11^th^	14'20'' /10^th^	08'37'' /3^rd^	-	-	05'37'' /2^nd^	11'50'' /5^th^	06'59'' /2^nd^	08'51'' /3^rd^	06'13'' /1^st^	08'17'' /3^rd^	-
Correct answers/ Attempts	1/11	1/2	1/5	4/9	2/13	2/11	1/3	0/12	0/11	2/5	2/7	3/7	3/9	2/2	2/5	26/112 (M*-1.7/7.5) (23.2% correct)
Asked demonstration	Yes	No	Yes	No	Yes	Yes	No	Yes	Yes	No	No	No	No	No	No	6 (40%)
Recently used Kinect/Xbox	No	No	No	No	No	No	No	No	No	No	No	No	No	No	Yes	1 (6.7%)
Prescription glasses	Yes	No	No	No	No	No	No	No	No	No	No	No	No	Yes	No	2 (13.3%)
Right/Left Handed	R^†^	L^‡^	R^†^	R^†^	R^†^	R^†^	R^†^	L^‡^	R^†^	R^†^	R^†^	L^‡^	R^†^	R^†^	R^†^	R^†^-12 (80%)/ L^‡^-3 (20%)

* M=Mean;

† R=Right handed;

‡ L=Left handed

**Figure 3:** Performance of the undergraduate students who took part in the VIDA-Nursing v.1.0 Simulation. Ribeirão Preto, SP, Brazil, 2018

Performance	1	2	3	4	5	6	7	8	9	10	11	12	13	14	15	Total
Duration of 1^st^ environment	01'25''	01'04''	01'31''	01'15''	02'06''	02'45''	02'19''	02'00''	03'30''	01'25''	02'02''	01'21''	01'10''	01'40''	01'45''	27'18'' (M*-01'49'')
Duration of simulation	10'47''	12'55''	08'00''	09'16''	18'32''	16'30''	25'37''	20'37''	17'09''	22'22''	08'52''	37'00''	16'09''	06'29''	24'49''	255'04'' (M*-17'00'')
Duration/first attempt	10'35'' /4ª	12'41'' /7ª	06'12'' /2ª	06'32'' /3ª	08'57'' /3ª	07'04'' /1ª	09'08'' /3ª	18'17'' /9ª	14'09'' /7ª	06'50'' /5ª	06'53'' /3ª	30'43'' /18ª	04'29'' /1ª	05'51'' /2ª	24'39'' /19ª	-
Correct answers/ Attempts	1/4	1/7	2/3	2/5	3/12	4/5	3/15	1/11	2/9	10/23	2/4	2/21	2/8	1/2	1/19	37/148 (M*- 2.5/9.9) (25% correct answers)
Asked demonstration	Yes	Yes	No	No	No	No	No	Yes	Yes	No	No	Yes	No	No	Yes	6 (40%)
Recently used Kinect/Xbox	No	No	Yes	No	No	No	No	Yes	No	Yes	Yes	Yes	Yes	No	No	6 (40%)
Prescription glasses	Yes	Yes	No	No	Yes	No	No	No	No	No	Yes	No	No	No	No	4 26.7%)
Right/Left Handed	R^†^	L^‡^	R^†^	R^†^	L^‡^	R^†^	R^†^	R^†^	R^†^	R^†^	R^†^	R^†^	R^†^	R^†^	R^†^	R^†^13 (86.7%)/ L^‡^-2 (13.3%)
Semester	8^th^	8^th^	8^th^	8^th^	8^th^	8^th^	8^th^	8^th^	8^th^	8^th^	4^th^	4^th^	6^th^	7^th^	10^th^	-

* M = Média;

† D = Destro;

‡ S = Sinistro

**Figure 4:**
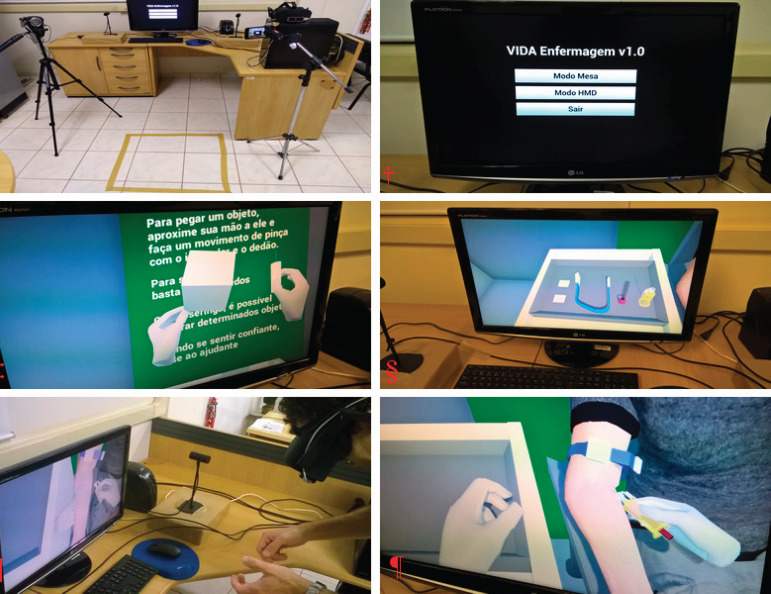
VIDA-Nursing v1.0 ^*^Configuration of equipment; ^†^Initial screen; ^‡^Ambiance setting; ^§^Material;^||¶^Venipuncture performance

The configuration of the equipment used in the simulation, initial screen, ambience setting, material, and procedure are portrayed in Figure 4.

The workers assessing the VIDA-Nursing v1.0 simulator considered 79.6% of the assessed items to be valid, while the students considered 66.7% of the items valid.

## Discussion

Technological resources in the teaching of nursing are developing constantly, however, the use of immersive VR simulation in the implementation of these strategies is seldom explored. One study analyzing the contributions of digital educational technologies in the teaching of nursing, reports that only two (6.7%) out of 30 studies involved the use of virtual reality^(^ 51 ^)^.

An analysis of the literature^(^ 52 ^)^ addressing strategies used in the teaching of the peripheral venipuncture procedure identified three simulations developed with VR, without using immersion resources: Virtual I.V. simulator (Laerdal Medical), CathYes AccuTouch System simulator (Immersion Medical, Inc.), and IV SIM simulator (AR-vision, Daejeon, Republic of Korea). Most of these studies report positive results in the use of simulators, though, those comparing traditional methods with virtual simulators did not present significant results but show that the combination of strategies, that is, virtual simulations with traditional methods, is indicated for the teaching of the peripheral venipuncture procedure. These simulators have a haptic unit in which users perform the peripheral venipuncture and see the result in computer virtual simulation. The haptic unit is an interesting resource for tactile interaction during simulations, however, these simulators, in addition to being costly, employ a mouse during part of the interaction.

The Leap Motion gesture sensor associated with HMD Oculus Rift was used in the development of the VIDA-Nursing v1.0 simulator. The gesture sensor is a screening device that captures the movement of hands and fingers with high precision^(^ 53 ^)^. The Head-mounted Display (HMD) enables the total immersion of users in an artificial environment through two liquid crystal displays that allow for stereoscopic vision^(^ 13 ^)^. Using these devices together is beneficial to facilitate programming and also because of the great potential of these two devices when used together with the sensor system and users’ immersion. Having the hands free to manipulate objects in the virtual environment and reproducing the environment with stereoscopy visualization using HMD allows users to experience a very realistic artificial world, an essential characteristic to develop hand-eye coordination that is part of most invasive procedures used in the health field.

In general, the VIDA-Nursing v1.0 simulator was considered a valid and promising tool to teach the procedure of vacuum blood collection in adults among undergraduate nursing students being introduced to this subject and technique. This simulator is expected to be used to teach this procedure after implementing suggestions and improvements needed for future versions. Studies addressing the development of immersive VR strategies have reported positive results in the field of teaching in the health field, especially in the training of specific procedures^(^ 41 ^,^ 54 ^)^.

In the workers’ assessment, nine items in the Movement Simulation Reality Aspect were considered to require revision (Q33, Q34, Q37, Q40, Q41, Q43, Q44, Q45 and Q46). The assessment concerning the User-friendly Aspect shows that two items (Q52 and Q53) need to be revised, while item Q54 was well rated.

The students’ assessment shows that 18 items require revision, five are related to the Visual Aspect (Q7, Q8, Q9, Q10, and Q15), one item in the Interactive Aspect (Q33), nine items in the Movement Simulation Reality Aspect (Q37, Q39, Q40, Q41, Q43, Q44, Q46, Q47, and Q48) and three items in the User-friendly Aspect (Q52, Q53, and Q54).

The items in the Visual Aspect, which according to the students, require review (Q7, Q8, Q9, Q10, and Q15), that is, Difficulty in visualizing the needle (Q7), Dry cotton (Q8) and Cotton soaked with alcohol (Q9), may be related to the system’s limited resolution, especially concerning the needle bevel. We believe this assessment may have been influenced by the fact that four students wore prescription glasses to see nearby objects. The workers, however, did not consider necessary to review any items in the Visual Aspect. Oculus Rift has a limitation for those wearing prescription glasses to correct hyperopia and a strategy to minimize users’ difficulty of visualization is to use a simulator version in which objects are presented on a scale larger than reality. The students were not familiar with the model of the tourniquet (Q10) used in the simulator and some students perceived the Tray (Q15) to be larger than reality. All these items can be improved by refining the modeling of virtual objects. Additionally, configuring the simulator according to each participant’s height before beginning the simulation can also better meet the specificities of users.

The items in the Interactive Aspect that need revision include the precision of the device’s movements (Q33), the space required to perform the puncture movement (Q34), Vein puncture (Q40), Insertion of the vacuum tube into the adapter (Q41), Removal of the blood-filled tube from the adapter (Q44), Needle removal (Q46), Compression of the puncture site with cotton (Q47), and Placement of the intermediate on the tray (Q48). All the items are related to difficulties in performing fine movements (at the time of puncturing the vein and manipulating objects) and configuration of the space at the time of the puncture. These may be related to a limitation of the Leap Motion to capture some movements if their configuration is not well standardized^(^ 53 ^)^. There are, however, ways to improve the system’s calibration, such as modeling a larger caliber vein to facilitate the access of users and minimize the problem.

The item Space to move at the time of performing the puncture (Q34) generated doubts among some participants, even though details were provided in the assessment form and during the experiment. This item refers to the space available for the participants to move around physically, however, some participants understood it referred to the virtual environment. Therefore, this item will be revised.

Regarding the Movement Reality Simulation Aspect, improvements were suggested to the items that refer to the Arm tourniquet (Q37 and Q43), which in this first experiment was programed to be performed with one of the hands only and will be reconfigured for two hands. Additionally, Tighten skin with the non-dominant hand (Q39) was restricted to one site restricting the puncture area. Assessment of the Homogenization of the blood sample (Q45) suggests that the blood inside the tube follows the rotating movement of the tube. Suggestions for the Compression of puncture site with cotton (Q47) and Placement of the intermediate on the tray (Q48) are that blood drips from the site in case it is not sufficiently compressed and that the intermediate does not return positioned with the needle upward, as it does at the beginning of the simulation; rather it should appear lying on the tray or with the possibility of being discarded into a sharp container. Another measure to improve these stages is the configuration of the structures in the virtual environment so that there is no overlap among them, hence users do not visualize one object entering another.

The implementation of the improvements previously mentioned is expected to solve the issues in the User-friendly Aspect (Q52, Q53, and Q54). Other options to make the simulator more user-friendly include: making a version for left-handed users or enable them to be free to choose their left or right hand at the time of tightening the skin when performing the puncture procedure. The system in this current version recognizes the left hand tightening the skin and if a left-handed user tights the skin with the right hand and punctures the vein with the left hand, the system indicates hands are inverted and impedes the simulation to continue. Left-handed participants performed the simulation with their right hands. Only one, out of the five left-handed participants, did not complete the procedure, showing the simulator is easy to use and will be useful for undergraduate nursing students to learn the technique.

One of the aspects to be considered in the use of teaching technologies is how much technology is currently present in people’s lives. Between the two groups, workers reported having less frequent contact with VR devices while some students reported having previously used these devices applied in teaching settings, which may have influenced the groups’ performance and interest in participating and assessing the simulator.

Only two, out of the 30 participants were not able to complete the procedure though these were workers who draw blood samples using a vacuum tube almost daily. The workers with extensive professional experience who do not perform this procedure daily were able to perform the simulation more easily; similar to those who had no practice in the technique, as was the case of the students.

Because the group of more experienced workers had performed this procedure numerous times, they wanted to see a simulation of patients’ behavior (especially verbal communication) in addition to the procedure, to be also rapid and individualized, considering that each worker has a specific manner to perform the procedure according to recommended guidelines. When we analyze operant behavior, as proposed by Skinner^(^ 49 ^)^, lack of behavior in the simulator, as workers expected, generated a negative reinforcement, that is, they were not supported to carry on with the procedure. For students or inexperienced workers, facing difficulties to complete the simulation up to its end was positive reinforcement, a challenge for the users to end the simulation only after completing all the stages. Additionally, the less experienced, the greater the positive reinforcement on one’s behavior to complete the procedure, as students’ scores show. The students’ mean number of correct answers was higher (2.5 correct answers/9.9 attempts) than that obtained by the workers (1.7 correct answers/7.5 attempts). Another operant behavior response was that the longer a participant interacted with the simulator, the better his/her performance concerning the duration of use.

In addition to the various possibilities to make the simulator a more interactive and motivating tool among students, there are the priority items that need to be addressed in the next version: to add new devices to make the virtual simulation environment more adaptable to the students’ environment such as the use of prescription glasses with the immersion device for those who need vision correction and adjust the system to be used by both left- and right-handed individuals. These and other improvements can be addressed in future versions as this study progresses and new demands and opportunities are identified.

Feedback will be provided to users in the simulator’s final version at the end of the simulation, presenting adverse events that took place during the procedure to promote a positive reinforcement for the learning of users. Even though the current version provides little feedbacks to users, promising effects of positive reinforcement were identified during its use.

Among this study’s potential biases and limitations, there is the fact that data collection indicated that, at the end of the day, the gesture sensor seemed to have its performance decreased, requiring pauses between simulations to improve its performance. The assessment form was considered comprehensive but required time and attention from the participants during its completion. Having left-handed participants as well as individuals wearing prescription glasses to be able to see nearby objects - without previously adapting the system - may have influenced how these individuals’ assessed the simulator. These limitations had not been foreseen.

## Conclusion

The development of the VIDA-Nursing v1.0 simulator revealed that obtaining a complete procedure simulator is a complex task. Numerous technological resources need to be used and incorporated during its implementation, which will be only achieved in the course of this study, by testing and improving research, so that we will be able to achieve a final product that can be incorporated as a teaching resource in nursing schools and meet the needs of this target population regarding the learning of this procedure.

The 14 steps performed during the simulation were assessed through 54 items. Even though according to the participants, approximately one-third of the items required revision, the VIDA-Nursing v1.0 simulator was considered a promising tool to teach the procedure of blood collection using a vacuum tube in adult patients. The reason is mainly that it can be combined with resources currently used to teach this procedure to undergraduate nursing students and therefore, provide better training to students so they develop the competencies needed to care for patients during supervised training and later, in their professional practice.
